# Science-based safety assessment of genetically modified DP915635 maize

**DOI:** 10.1080/21645698.2024.2439116

**Published:** 2024-12-31

**Authors:** Kent Brink, Tim Gunderson, Matthew Harmon, Kristine LeRoy, Margit Ross, Anitha S.C., Brian Stolte, John Zhang

**Affiliations:** a Corteva Agriscience, Regulatory Science, Johnston, IA, USA; b Corteva Agriscience, Regulatory Science, Hyderabad, India

**Keywords:** Environmental risk assessment, genetically modified, insecticidal protein, regulatory guidance, safety assessment

## Abstract

Genetically modified maize event DP-915635-4 expressing the IPD079Ea protein was developed to control corn rootworm damage to maize plants. Utilizing a modernized safety assessment model published by CropLife International (CLI), the safety of DP-915635-4 maize was assessed. The CLI core studies included were molecular characterization of the inserted DNA, expression and characterization of the expressed IPD079Ea protein, and its safety for both food and feed use and the environment. No hazards were identified for human or animal consumption of DP-915635-4 maize containing the IPD079Ea protein, indicating that supplementary studies were not necessary. An environmental risk assessment was performed to characterize any potential impacts to non-target organisms, the results of which established that DP-915635-4 maize is unlikely to result in unreasonable adverse effects to non-target organisms. This case study shows that the modernized safety assessment is effective at demonstrating the safety of genetically modified crop plants.

## Introduction

Extensive experience and knowledge has supported the economic/environmental benefits and food/feed safety of genetically modified (GM) crops over the last three decades.^1^ Expanding upon this knowledge, CropLife International (CLI) recently published a series of papers describing a modernized science-based approach for assessing the safety of GM crops that make use of the experience and scientific advancements made over the last 30+ years.^2–5^ Specifically, this stepwise approach recognizes our centuries-long experience in safe crop breeding and the comparative risk between traditional breeding and transgenesis. For food and feed safety, the CLI safety assessment applies an initial weight-of-evidence to three core assessments: molecular characterization, protein expression and characterization, and protein safety.^3,5^ Following this stepwise approach, data from the core studies support the assessment of potential hazard of the protein. If a hazard is identified, protein expression data support worst-case exposure estimates which are then used to evaluate risk. Depending on the outcome, further hypothesis-driven studies may be indicated.

GM crops which include pesticidal proteins may also require an environmental risk assessment (ERA) used to evaluate potential pathways to environmental harm.^2^ In the case of proteins like those previously deployed (e.g., Cry or Vip proteins), this may be based on familiarity with previous traits. In the case of newly developed proteins lacking familiarity, the potential for environmental harm was characterized which resulted in an assessment of potential risk to non-target organisms (NTO) and environmental fate. Potential for adverse effects to NTOs may be ascertained by analyzing the spectrum of activity of the protein against representative organisms, while environmental fate may be determined by analysis of protein stability in soil.

Considering this approach as an example, the safety of GM maize event DP-915635-4 (DP915635), which provides protection from susceptible below-ground insect pests and confers tolerance to glufosinate herbicide, is examined. DP915635 maize expresses the IPD079Ea protein, derived from the fern *Ophioglossum pendulum*, which has been developed to help control corn rootworm (*Diabrotica* spp.),^6^ the most economically important maize insect pest in North America.^7^ DP915635 maize also expresses the phosphinothricin acetyltransferase (PAT) protein that imparts tolerance to glufosinate herbicide^8^) and the phosphomannose isomerase (PMI) protein used as a selectable marker for *in vitro* recovery of transgenic events following transformation. Both the PAT and PMI proteins have been widely present in GM crops for many years and have a history of safe use.^8,9^ Using the CLI modernized assessment,^2,3^ the food and feed safety assessment for DP915635 maize is presented using relevant core data on the molecular characterization, protein expression and characterization, and protein safety supporting the IPD079Ea protein are presented. The ERA of DP915635 maize leverages problem formulation and demonstrates the risk associated with cultivation of DP915635 maize is low.

## Methods and Materials

### Molecular Characterization

DP915635 maize was created by site-specific integration using two sequential transformation steps to 1) insert an integration site sequence (referred to as a “landing pad” sequence) at a specific location in the maize genome using microprojectile bombardment and a CRISPR-Cas9-mediated targeted insertion process, and 2) insertion of the intended expression cassettes into the landing pad via recombination following *Agrobacterium*-mediated transformation using a plasmid carrying these genes.

Under the CLI modernized model, core studies to molecular characterization of the transgenic crop include: 1) number of insertion loci and inserts per locus, 2) presence or absence of unintended sequences (e.g., plasmid backbone), 3) sequence of the inserted DNA, and 4) stability of inserted DNA across multiple generations.

#### Sequencing of Insert and Its Flanking Genomic Regions of DP915635 Maize

Genomic DNA (gDNA) was extracted from a pool of finely ground leaf tissue from ten DP915635 maize plants using a urea lysis buffer, purified using phenol/chloroform/isoamyl alcohol (25:24:1) separation, RNase treatment, DNA precipitation, and spooling. Overlapping PCR fragments spanning the insert and the 5’ and 3’ flanking border regions were amplified from the gDNA of DP915635 maize and cloned into pCR™Blunt IITOPO™ vectors (Invitrogen). The resulting plasmid DNA was sequenced by the Sanger sequencing method (Eurofins) in both forward and reverse directions to cover every nucleotide at least six times. The sequence data was analyzed and assembled in Sequencher® Version 4.8 software (Gene Codes Corporation) to determine the final consensus sequence for the DP915635 insert and its flanking genomic regions.

#### Southern Blot Analysis for Insertion Number, Stability Across Generations, and Unintended Plasmid Sequences

Southern blot analysis was conducted as previously described.^10^ gDNA was isolated from DP915635 and non-GM control plants using a high-salt buffer (2.0 M NaCl, 100 mm Tris-HCl pH-8.0, 50 mm of sodium salt of EDTA, and 100 mm sodium metabisulphite) followed by potassium acetate and isopropanol precipitation, RNase treatment, and reprecipitation using sodium acetate and ethanol. Samples (10 µg) were digested with the appropriate restriction enzyme (Thermo Fisher Scientific), separated on an agarose gel along with DIG labeled DNA Molecular Weight Marker III and VII (Roche) to serve as size standards, transferred to a nylon membrane (GE Healthcare), and fixed by UV crosslinking. Digoxigenin (DIG) labeled DNA probes were labeled by PCR using the appropriate plasmid template to incorporate labeled DIG-11-dUTP nucleotide (Roche) into the probes. Probes were hybridized using DIG Easy Hyb™ (Roche) and bands detected using the DIG Wash and Block Buffer Set and CDP-Star Chemiluminescent Nucleic Acid Detection System (Roche).

#### Insertion Copy Number

gDNA from the T1 generation of DP915635 maize was digested with the restriction enzyme PvuII, which has a single recognition site within the DP915635 insert. Following electrophoresis and transfer, the DNA fragments on the membrane were hybridized with probes to all the genetic elements in the DP915635 insert.

#### Stability of the Insertion Across Generations

gDNA isolated from five generations of DP915635 maize (T1, T2, T3, T4, and T5 generations) and non-GM control maize was digested with the restriction enzyme PvuII and hybridized with probes for the *pmi*, *mo-pat*, and *ipd079Ea* genes. The presence of equivalent bands across all generations would demonstrate the stability of the inserted DNA during the breeding process.

#### Investigation of Unintended Sequences from Plasmids

gDNA isolated from the T1 generation of DP915635 maize and control maize was digested with selected restriction enzymes and hybridized with probes designed to cover the sequences of all plasmids used in the creation of DP915635 maize that were not intended to be incorporated in the DP915635 insert (e.g., plasmid backbone or genes utilized for the site-specific integration process or to improve transformation and plant regeneration response). Detection of unexpected bands in the DP915635 samples (not resulting from homologous sequences in the insertion or endogenous to the maize genome) would indicate the presence of additional inserted DNA from the plasmids that was not intended to be in the DP915635 maize genome.

### Protein Characterization and Analysis of Expression Levels in Maize

Under the CLI modernized model, core studies to protein characterization of newly expressed proteins in the transgenic crop are: 1) molecular weight, 2) amino acid sequence, and 3) protein function. Protein characterization data are also generated for any surrogate protein (e.g., microbe produced) used to support the safety assessment to determine if it is sufficiently similar to the plant-produced protein for its intended use. Protein expression levels in key crop tissues are also determined to estimate worst-case exposure levels where a hazard is identified and/or where exposure-based doses or concentrations are used in non-target toxicity tests.

#### Purification of Maize-Derived Protein

The IPD079Ea protein was extracted from lyophilized maize tissue collected from field-grown plants using phosphate-buffered saline containing polysorbate 20 (PBST) extraction buffer over several batches targeting a ratio of 1 g of tissue to 20 ml of extraction buffer. The sample extract was then filtered and clarified by centrifugation. The IPD079Ea protein was partially purified by immunoaffinity chromatography using columns prepared by coupling an IPD079Ea monoclonal antibody to AminoLink Plus Coupling Resin. Select IPD079Ea elution fractions from the immunoaffinity purification columns were neutralized with 0.1 CV 1 M Tris, pH 8 and were concentrated and buffer exchange using a centrifugal concentrator (30K Vivaspin; Sartorius).

#### Molecular Weight

The partially purified maize-derived IPD079Ea protein and a purified microbe-derived IPD079Ea protein control were prepared and diluted as needed in 4X NuPAGE LDS sample buffer and 10X NuPAGE sample reducing agent to obtain a final concentration of 1X NuPAGE LDS sample buffer and 1X NuPAGE sample reducing agent using ASTM (American Society for Testing and Materials) Type I water (referred to as water) as needed for diluent. Coomassie staining and western blot analysis followed the methods used to analyze the microbe-derived IPD079Ea protein documented in Carlson et al.^11^

#### Amino Acid Sequence

Two IPD079Ea maize-derived protein bands were excised from two lanes of an SDS-PAGE gel and stored at ≤ −5°C. The protein in each gel slice was reduced with dithiothreitol (DTT), alkylated with iodoacetamide, and then subsequently digested separately with trypsin and chymotrypsin. The digested samples were analyzed by liquid chromatography-mass spectrometry (LC-MS) as described for the microbe-derived IPD079Ea protein in Carlson et al.^11^ except a newer version of the Mascot and GPMAW software was used for analysis of the plant-derived IPD079Ea samples.

#### N-Terminal Sequencing

The maize-derived IPD079Ea protein was analyzed by Edman sequencing as described in^11^ for the microbe-derived IPD079Ea protein.

#### Protein Equivalence Between the Microbe- and Maize-Derived Proteins

Production, purification, molecular weight determination, and sequencing were described previously for the surrogate microbe-expressed protein used for non-target testing.^11^ Functional equivalence between the maize-derived and microbe-derived IPD079Ea protein was determined via bioassay with western corn rootworm (*Diabrotica virgifera virgifera*) neonate larvae. Assays were conducted in 96-well bioassay plates containing 200 µl of an agar-based rootworm diet. The maize-derived and microbe-derived proteins were surface applied with 25 µl per well to target concentrations of 1.56, 3.13, 6.25, 12.5, and 25.0 µg/cm^2^ in a 50 mm Tris, pH 8, buffer. Plates were dried and one neonate larva was placed in each well. Treatments were arranged in a randomized complete block design with a total of three blocks. Each block consisted of two 96-well bioassay plates and contained eight replicates from each treatment. The plates were covered with heat-sealing film and ventilated with one small hole above each well. Each treatment was provided to 24 individual western corn rootworm larvae. The bioassay was held at 21°C, 65% relative humidity, and continuous dark. After 7 days, mortality was assessed. LC_50_ values and 95% fiducial limits of the maize-derived protein and the microbe-derived protein were estimated using the PROBIT procedure of SAS software (Version 9.4). The probability of mortality was modeled as a function of the base-10 logarithm of the concentration. Since the observed baseline mortality was non-zero, a natural mortality was estimated with OPTC option.

#### Analysis of Protein Expression Levels in Maize

Field tissue production and sample handling of DP915635 maize have been previously described.^12^ A validated ELISA method (unpublished) was used to evaluate the expression of the IPD079Ea protein in root, leaf, pollen, forage, and grain at various growth stages (Table 1) using samples from six field sites in Iowa, Illinois (two sites), Nebraska, Pennsylvania, and Ontario.

**Table 1.** Mean expression and range across field sites for IPD079Ea protein in DP915635 maize.

Growth Stage and Tissue Type	Mean IPD079Ea Expression (ng/mg)	Range of Expression (ng/mg)
V6 root	16	9.9–26
V9 root	9.2	0.72–30
R4 root	1.2	0.28–2.7^b^
V9 leaf	0.83	0.33–1.6
R1 leaf	0.16^a^	<0.14–0.29^b^
R4 leaf	<0.14	<0.14
R1 Pollen	0.95	0.58–1.3
R4 Forage	0.25	0.086–0.46
R6 Grain	0.18	0.075–0.36

Samples of the indicated tissue type and growth stage^13^ from six field sites were analyzed by ELISA for IPD079EA protein concentrations and the means calculated. Unless otherwise indicated, the mean is calculated using a total of 24 samples. A “lower than” symbol (<) indicates that samples fell below the lower limit of quantification (LLOQ) for the assay.

^a^Some, but not all, sample results were below the LLOQ. A value equal to half the LLOQ value was assigned to those samples to calculate the mean.

^b^Sample integrity was compromised during sample preparation; results from 20 leaf (R1 growth stage) samples and four root (R4 growth stage) samples were considered invalid and not reported.

### Protein Safety Assessment

The CLI modernized model for the safety assessment of transgenic crops focuses on the newly expressed proteins and investigates the following core information: 1) history of safe use of the newly expressed proteins and the source organisms for these proteins, 2) bioinformatic investigations comparing the amino acid sequence of the newly expressed proteins with that of toxins and allergens, and 3) knowledge of the mode of action of the newly expressed proteins. If results from core studies are not conclusive for safety, then additional hypothesis-driven supplementary studies might be indicated.

#### History of Safe Use of the IPD079Ea Protein and Its Sources Organism

The history of safe use of the IPD079Ea protein and its source organism has been described previously.^11,14^ Briefly, the history of safety for the fern, *Ophioglossum pendulum*, source organism for the IPD079Ea protein and ferns in general was evaluated, along with the distribution of proteins in the IPD079Ea protein family across fern species with a history of safe use.

#### Bioinformatics

Bioinformatic data supporting the IPD079Ea protein has been reported previously.^11^ Briefly, the amino acid sequence of the IPD079Ea was used to query the Corteva Agriscience toxin database using the BLASTP search algorithm with an *E*-value setting of 10^−4^. The sequence was also used to query the COMPARE allergen database using two approaches: 1) A ≥ 80 amino acid sliding-window search looking for >35% identity, and 2) A search looking for eight amino acid identical contiguous matches.^15^

### Environmental Risk Assessment

The environmental risk assessment conducted for DP915635 focused on potential non-target effects (potential exposure and hazard) of the corn rootworm-active IPD079Ea protein and its potential persistence in the environment. The risk assessment framework involves 1) evaluating the spectrum of activity against insect pests, pollinators, beneficial insects, insectivorous birds, and granivorous mammals, and 2) evaluating protein stability in soil.^2,6,16^

The activity of the IPD079Ea protein against ten beetle (coleopteran) species [*Diabrotica virgifera virgifera* (western corn rootworm), *Diabrotica undecimpunctata* (southern corn rootworm), *Leptinotarsa decemlineata* (Colorado potato beetle), *Tenebrio molitor* (mealworm), *Zophobas morio* (superworm), *Tribolium castaneum* (red flour beetle), *Epilachna varivestis* (Mexican bean beetle), *Hippodamia convergens* (convergent lady beetle), *Coleomegilla maculata* (spotted lady beetle), and *Dalotia coriaria* (greenhouse rove beetle)] and four moth/butterfly (lepidopteran) species [(*Ostrinia nubilalis* (European corn borer), *Helicoverpa zea* (corn earworm), *Vanessa cardui* (painted lady), and *Cydia pomonella* (codling moth)] has been reported previously along with bioassay methods.^6^ Activity against additional arthropods, including: *Apis mellifera* (honeybee larvae and adults), *Folsomia candida* (springtail), *Daphnia magna*, *Chrysoperla rufilabris* (green lacewing), and *Pediobius foveolatus* (parasitic Hymenoptera) was investigated, along with activity against *Colinus virginianus* (Northern bobwhite quail).^16^ A 14-day mouse acute oral study with microbe-derived IPD079Ea has been previously reported.^11^

#### Persistence in Soil was Investigated as Follows

Three soil types (loam, sandy loam, and silt loam) were spiked with aqueous solutions of microbe-derived IPD079Ea protein at 4,107, 3,595, and 3,879 ng/mg dry-weight, respectively. Control samples of each soil type were also spiked with water for use in the bioassays. These freshly spiked samples were used as day-zero (day 0) samples for western blot analysis and insect (western corn rootworm) bioassay. The remaining tubes were incubated at 20°C and 80% relative humidity.

Additional samples of each IPD079Ea spiked soil type were collected on days 1, 2, 3, 4, 7, and 14, and extracted for western blot analysis. On the day of collection, IPD079Ea-spiked soil samples (~250 mg) were extracted in 600 µl of 1X lithium dodecyl sulfate (LDS)/DTT sample buffer (25% 4X NuPAGE LDS Sample Buffer, 10% 10X NuPAGE Sample Reducing Agent containing DTT, and 65% water). In addition, on the day of soil-spiking, a sub-sample was prepared for SDS-PAGE by combining 65% IPD079Ea protein, 25% 4X LDS sample buffer, and 10% reducing agent. All the samples were heated at 90–100°C for 5 min and stored frozen at −80°C. Prior to SDS-PAGE, the samples (two per timepoint for each IPD079Ea-spiked soil type) were thawed and heated at 90–100°C for 5 min. After diluting, the samples were loaded into 4–12% Bis-Tris gels along with a microbe-derived IPD079Ea protein control sample and pre-stained protein molecular weight markers (Precision Plus Protein Dual Xtra Standards). SDS-PAGE and western blot were performed as described previously^11^ except an IPD079Ea polyclonal antibody was used as the primary antibody.

In addition to the western corn rootworm (*Diabrotica virgifera virgifera*) bioassay initiated on day 0, a bioassay was initiated on day 7. Bioassays were conducted by incorporating soil samples into a modified Stonefly Heliothis artificial diet. On each day of diet preparation, three randomly selected aliquots of the IPD079Ea protein-spiked soil and three control soil samples for each soil type were removed from the environmental chamber, pooled by treatment, and mixed with carrier at approximately 20% soil by dry weight of carrier. Water was added to the dry soil-carrier mix at 2.51 ml water to 1 g carrier. A single neonate rootworm was added to each well of 24-well bioassay plates containing approximately 300 µl of freshly prepared diet and plates were covered with vented heat-sealing film and held at 21°C, 65% relative humidity in continuous dark for 7 days. Treatments were arranged in a generalized randomized block design with a total of ten blocks. Each block consisted of a 24-well bioassay plate and contained three replicates from each treatment. Each treatment was fed to 30 larvae which were refed on day 4 of each bioassay with soil aliquots that had been incubated an additional 4 days after the initiation of the bioassay. After 7 days, mortality was assessed.

## Results

### Molecular Characterization

**Figure 1.**
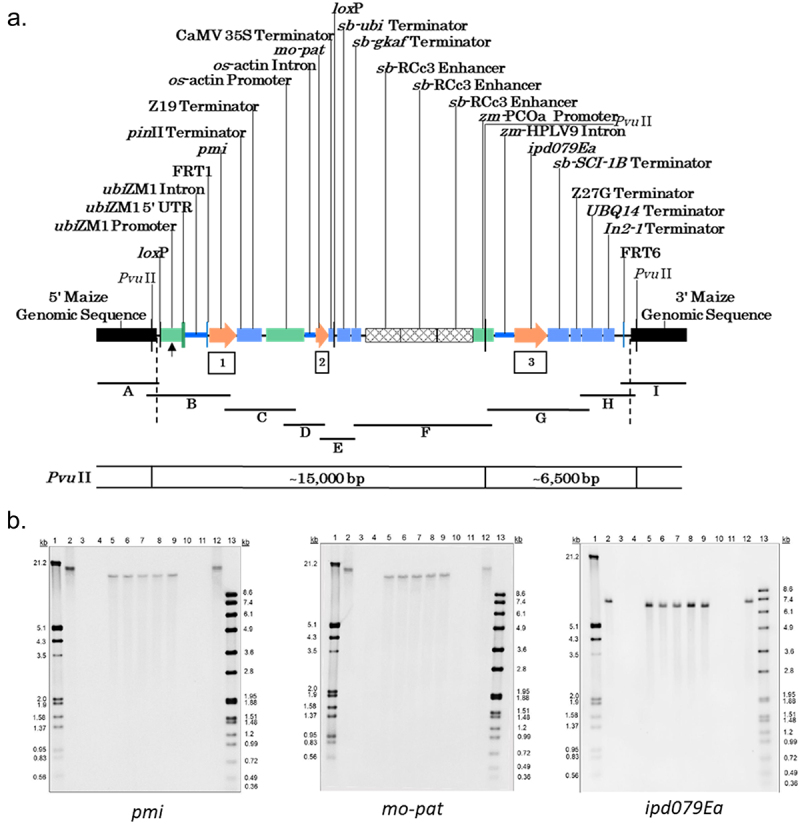
Molecular characterization of DP915635 maize. a) Map of the inserted DNA in the DP915635 maize genome following the SSI integration of the intended gene cassettes. The ends of the DNA insertion are indicated by dashed vertical lines and flanking maize genomic regions are represented by the horizontal black rectangular bars. The black lines below the map indicate the PCR fragments used for Sanger sequencing of the DNA insert and genomic flanking sequences. The arrow shows the location of the single base change identified in the ubiZM1 promoter. Southern blot probes are shown by the boxes (1. *pmi*, 2. *mo-pat*, 3. *ipd079Ea*). *Pvu*II restriction sites are indicated with the size of the observed fragment shown below the map in base pairs (bp). b) Five-generation Southern blots digested with *Pvu*II and hybridized with the indicated probes. The presence of single and consistent bands in five generations indicates the presence of a single stably inherited DNA insert in DP915635 maize. Lane 1: dig-labeled DNA marker III; lanes 2 & 12: positive controls; lanes 3 & 11: PHR03 control maize; lanes 4 & 10: Blank; lane 5: DP915635 maize T1 generation; lane 6: DP915635 maize T2 generation; lane 7: DP915635 maize T3 generation; lane 8: DP915635 maize T4 generation; lane 9: DP915635 maize T5 generation; lane 13: dig-labeled DNA marker VII.

The DP915635 insert was confirmed to have the expected sequence except for a single nucleotide change (A to C) in the *ubi*ZM1 promoter driving PMI expression. Southern analyses demonstrated the presence of a single insertion in DP915635 maize and confirmed equivalent single bands from hybridization to five generations of DP915635 maize with each of the *pmi*, *pat*, and *ipd079Ea* probes, indicating stability of a single insertion across multiple generations (Figure 1). Southern blot analysis with probes to unintended plasmid sequences did not detect any unexpected presence of such sequences not intended to be transferred to the DP915635 maize genome. Thus, DP915635 should express only the intended proteins.

### Maize-Expressed Protein Characterization and Equivalence with the Microbe-Derived Surrogate Protein

**Figure 2.**
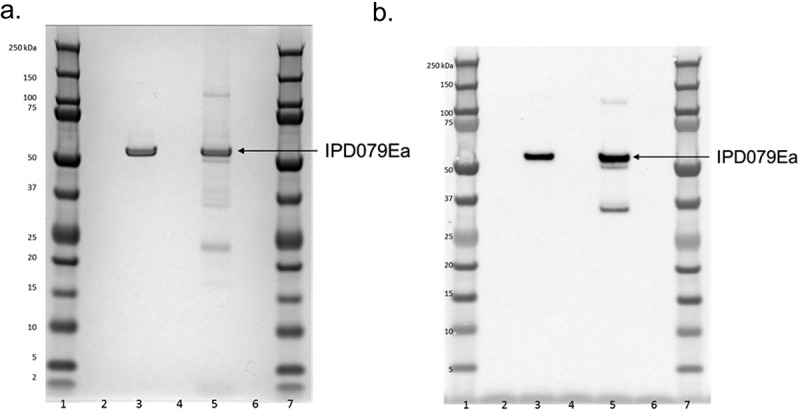
Analysis of the DP915635 Maize-derived and microbe-Derived IPD079Ea proteins. a) SDS-PAGE. b) Western blot. Lanes 1 & 7: pre-stained protein molecular weight marker (included to provide a visual estimate that migration was within the expected range of the predicted molecular weight); lanes 2, 4 & 6: 1X LDS/DTT sample buffer blank; lane 3: microbe-derived IPD079Ea protein (1 µg for SDS-PAGE, 10 ng for western blot); lane 5: DP915635 Maize-derived IPD079Ea protein (diluted 1:2 for SDS-PAGE, 1:100 for western blot).

**Figure 3.**
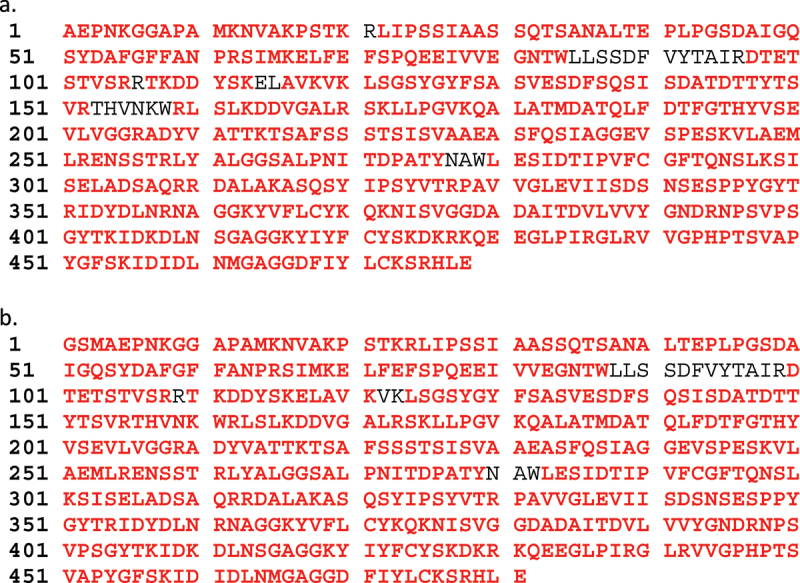
Identified tryptic and chymotryptic peptide amino acid sequence of IPD079Ea protein samples by LC-MS analysis. a) Maize-derived IPD079Ea. b) Microbe-derived IPD079Ea. Red type indicates IPD079Ea peptides identified using LC-MS analysis. Amino acid residue abbreviations: alanine (A), cysteine (C), aspartic acid (D), glutamic acid (E), phenylalanine (F), glycine (G), histidine (H), isoleucine (I), lysine (K), leucine (L), methionine (M), asparagine (N), proline (P), glutamine (Q), arginine (R), serine (S), threonine (T), tryptophan (W), tyrosine (Y), and valine (V).

Coomassie staining of the SDS-PAGE gel and western blot analysis demonstrated the DP915635 maize-derived IPD079Ea protein migrated as a predominant band consistent with the expected molecular weight of approximately 52 kDa and the microbe-derived IPD079Ea protein control (Figure 2). The matched peptides identified with the LC-MS analysis of the trypsin- and chymotrypsin-digested IPD079Ea protein accounted for 94.8% (453/478) of the expected IPD079Ea amino acid sequence (Figure 3). The Edman N-terminal amino acid sequence analysis of the maize-derived IPD079EA protein obtained no sequence data, suggesting the N-terminus of the protein was blocked. The N-terminal peptide was identified as AEPNKGGAPAMK from LC-MS analysis of the tryptic digestion of the protein. The results confirmed the absence of the N-terminal methionine as expected and blocked N-terminal alanine residue by acetylation. The IPD079Ea protein derived from DP915635 maize had the expected molecular weight, immunoreactivity, and amino acid sequence.

Both the microbe-derived IPD079Ea protein data presented by Carlson et al.^11^ and the data shown in Figure 2 demonstrated that the maize-derived and microbe-derived surrogate proteins have equivalent molecular weight. The expected sequences (Figure 3) were confirmed by LC-MS analysis with high coverage of 94.8% and 96% for the DP915635 maize-derived and microbe-derived IPD079Ea protein, respectively. The N-terminal amino acid sequence analysis of the microbe-derived IPD079Ea protein demonstrated that the sequence (GSMAEPNKGG) was consistent with the expected N-terminal amino acid sequence of the microbe-derived IPD079Ea protein (Figure 3). The microbe-derived IPD079Ea protein was expressed as a fusion protein with an N-terminal histidine tag to facilitate large-scale protein purification. The tag was cleaved by thrombin. Three additional N-terminal amino acid residues (glycine, serine, and methionine) remained following tag cleavage and removal. Except for the three additional N-terminal amino acids resulting from the fusion tag removal for production of the microbe-derived protein, the maize- and microbe-derived IPD079Ea proteins have the same sequences. In addition, bioactivity equivalence analysis was conducted with Western corn rootworm bioassays. LC_50_s for the surrogate microbe-derived protein ranged from 17.9 (5.58–115) to 26.2 (9.65–5130) µg/cm^2^ while the LC_50_ for the maize-derived protein was 6.93 (2.07–19.7) indicating similar potency with overlapping 95% fiducial limits.

### Ipd079ea Protein Expression in DP915635 Maize

Mean expression levels and the range across field sites for the IPD079Ea protein in DP915635 maize are found in Table 1.

### Protein Safety

While the history of safe use for the IPD079Ea protein itself and its *Ophioglossum pendulum* fern source organism are limited, safe consumption of other members of this fern genus has been documented.^11^ In addition, substances that have been identified as potentially causing adverse effects in vertebrates within ferns in general are non-proteinaceous, and members of the IPD079Ea family of proteins are found in fern species with a history of safe use.^14^ No biologically significant similarities were identified between the amino acid sequence of the IPD079Ea protein and that of known toxins or allergens. The activity of the IPD079Ea protein is limited to a subset of insect species^16^ and specific binding to gut receptor in the sensitive western corn rootworm has been observed, but such binding does not occur in several insensitive lepidopteran insect species.

### Environmental Risk Assessment: Spectrum of Activity and Soil Stability

Results of the spectrum of IPD079Ea activity evaluation and protein stability in soil are reported in Boeckman et al.^6^ and O’Neill et al.^16^ Estimated environmental concentrations (EECs) were calculated along with Margin of Exposure (MOE) to characterize the potential for environmental risk. The IPD079Ea protein has a narrow spectrum of activity and is not expected to result in unreasonable adverse effects on beneficial NTO populations at environmentally relevant concentrations.^16^

Of the ten coleopteran species evaluated for susceptibility to the IPD079Ea protein, only one species beyond rootworms (*Diabrotica virgifera virgifera* and *Diabrotica undecimpunctata*) showed greater than 20% mortality (*Epilachna varivestis*; Mexican bean beetle) at the highest concentrations tested (800 ng IPD079Ea/mg diet). None of the four lepidopteran species were found susceptible to the IPD079Ea protein up to 800 ng/mg,^6^ which is consistent with IPD079Ea binding to rootworm, *Diabrotica virgifera virgifera*, brush border membrane vesicles prepared from insect gut tissue but not to lepidopteran, *Ostrinia nubilalis* and *Helicoverpa zea* brush border membrane vesicles. Of the additional beneficial arthropod species evaluated, only *Chrysoperla rufilabris* (green lacewing) exhibited statistically significant mortality, and only at the highest concentration tested (500 ng IPD079Ea/mg diet). Furthermore, the MOE based on the No Observed Effect Concentration (NOEC) of 100 ng/mg of diet is 38.5-fold greater than the expected environmental concentration^16^ indicating negligible risk under field relevant conditions. Quail and mice exhibited no IPD079Ea treatment-related effects.^11,16^

The stability of IPD079Ea protein was assessed in three soil types: loam, sandy loam, and silt loam. After spiking the individual soils with IPD079Ea, degradation was observed by western blot analysis and insect bioassay within 7 days when incubated in each of the three soil types tested. Strong IPD079Ea bands on western blots were visible for all soil types on day 0, the day of IPD079Ea spiked into the soil. The IPD079Ea bands were visually undetectable within 7 days of incubation. Mortality for western corn rootworms fed soil spiked with IPD079Ea fortified diet on day 0 ranged from 43% to 79%. After 7 days of IPD079Ea incubation in soil, mortality was ≤10% across soil types. It may be concluded that IPD079Ea is rapidly degraded in this environmental matrix and unlikely to persist.

## Discussion

The data presented in this paper are focused on the safety of the IPD079Ea protein as described by the CLI modernized model. Characterization of the inserted DNA and newly expressed proteins establishes that the intended changes were made in DP915635 maize with results of the core studies supporting the safety of the IPD079Ea protein as expressed in DP915635 maize. PAT and PMI proteins, also expressed in DP915635 maize, while not a focus of this paper, have a demonstrated history of safe use and familiarity as a herbicide-tolerance trait and selectable marker, respectively.^8,9^

IPD079Ea expression data provides estimations of worst-case environmental exposure levels from DP915635 maize. No biologically significant similarities were found between the amino acid sequence of IPD079Ea and the sequences of toxins or allergens. The spectrum of activity for the protein within insects is limited and appears to be correlated with specific binding in the gut of susceptible species, as described. Activity of the IPD079Ea protein against surrogate beneficial species is very limited and expected environmental concentrations are at least an order of magnitude below the NOEC levels. From an environmental persistence perspective, as expected for a protein, the IPD079Ea protein was found labile in soil. Additionally, members of the IPD079Ea protein family are present across fern species with a history of safe use.^14^ In general, substances within ferns identified as potentially causing adverse effects in vertebrates are non-proteinaceous.

The science-based safety assessment performed following the modernized CLI model for the IPD079Ea protein found in DP915635 maize supports the conclusion that it is as safe as conventional maize for food and feed use and that it is also unlikely to result in adverse effects on the environment. No plausible pathway to cause harm was found in the food and feed safety assessment core studies. Under the CLI modernized model, this outcome is necessary and sufficient to determine the safety of DP915635 maize for food and feed use without further consideration of the conduct of supplementary studies. Additional studies would only be required if the weight-of-evidence from the core studies identified a potential hazard. Supplementary studies would then be hypothesis-driven based on the issue identified rather than dependent on a wide set of prescribed analyses that may not add value to the safety assessment. Potential for adverse effects from the consumption of IPD079Ea protein by NTOs was characterized, which found that DP915635 maize is not expected to cause unreasonable adverse effects to ecosystems in maize fields (O’Neill *et al*., 2024).

The core studies described for the IPD079Ea protein are sufficient to establish DP915635 maize is as safe as conventional non-GM maize, however, to fulfill current global regulatory requirements and to demonstrate agronomic and compositional comparability of DP915635 with non-GM maize, multiple supplementary studies were performed.^11,12^ Data and conclusions from these studies align with those from the core studies; both demonstrating that DP915635 maize is unlikely to result in adverse effects to human or animal health. This example of DP915635 maize and the expression of the IPD079Ea, PAT, and PMI proteins demonstrates that the core studies recommended by the CLI modernized model provide a framework for the food and feed safety assessment of GM crops.

## Statements and Declarations

Authors are employees of Corteva Agriscience^TM^ which has funded this research and develops the traits in and markets the GM Seed from those traits.
